# Constructing a DNA barcode reference library for southern herbs in China: A resource for authentication of southern Chinese medicine

**DOI:** 10.1371/journal.pone.0201240

**Published:** 2018-07-25

**Authors:** Lu Gong, Xiao Hui Qiu, Juan Huang, Wen Xu, Jun Qi Bai, Jing Zhang, He Su, Chu Mei Xu, Zhi Hai Huang

**Affiliations:** 1 Guangdong Provincial Hospital of Chinese Medicine, The Second Affiliated Hospital of Guangzhou University of Chinese Medicine, and China Academy of Chinese Medical Sciences Guangdong Branch, China Academy of Chinese Medical Sciences, Guangzhou, China; 2 Guangdong Provincial Key Laboratory of Clinical Research on Traditional Chinese Medicine Syndrome, Guangzhou, China; National Cheng Kung University, TAIWAN

## Abstract

Southern Chinese Medicine (SCM) is an important sect of Traditional Chinese Medicine (TCM) with its own special cultural style. Species identification is essential for TCM quality control because authentic herbs are possibly substituted with adulterants that would threaten the health of the public or even cause death. Here, we provided the first local reference DNA barcode library based on the second internal transcribed spacer (ITS2) for the molecular identification of SCM. A total of 1512 specimens of southern herbs representing 359 species were collected under the instructions and identification of taxonomic experts. Genomic DNA was extracted, and the PCR reaction proceeded according to standard procedures. After Sanger sequencing, sequence assembling and annotation, a reliable ITS2 barcode library with 1276 sequences from 309 species of Southern herbs was constructed. The PCR efficiency of the whole samples was 84.39%. Characteristics of the ITS2 barcode were analyzed, including sequence lengths and GC contents in different taxa. Neighbor-joining trees based on Kimura 2-Parameter (K2P) genetic distances showed a 67.56% successful rate of species identification with ITS2 barcode. In addition, 96.57% of species could be successfully identified at the genus level by the BLAST method. Eleven plant species were discovered to be cryptic. In addition, we found that there is an incorrect sequence existing in the public database, making a reliable local DNA barcode reference more meaningful. ITS2 barcodes exhibit advantages in TCM identification. This DNA barcode reference library could be used in Southern Chinese Medicine quality control, thus contributing to protecting public health.

## Introduction

China is one of the largest countries with land area worldwide and is also an important biodiversity hotspot. Various plants have been used to ease pain as Traditional Chinese Medicine (TCM) over long historical periods. In terms of the number of species, TCM represents by far the most important form of natural resource utilization [1]. Currently, TCM is widely used as complementary and alternative medicine in developing countries and has become increasingly popular in developed countries. It is reported that increasing global demand will boost the medicinal plant trade from approximately 120 billion USD to 7 trillion USD by the year 2050 [2]. However, adulteration in herbal products is a major threat to the therapeutic efficacy and safety of TCM [3–6]. Hence, species identification becomes a vital step in traded medicinal plants. Southern Chinese Medicine (SCM) is an inseparable part of TCM in China. Compared to the "North Chinese Medicine", SCM refers to Chinese medicine grown and produced in the southern area of China. The geographical range of SCM is not clear. Most literature involves Guangdong, Guangxi and Hainan provinces. In addition, some studies include Yunnan, Fujian, and Taiwan, and even Guizhou, Sichuan provinces. According to the survey, there were 3800 types of SCM, with 3500 southern plant species included [7]. Unique geography along with a more humid and warmer climate creates a special style of South Medicine culture compared to North Medicine in China [8]. For example, southern plants are always evergreen and do not change with seasons, making them easier to use. Southern Medicine has a broader mass base. Although not exactly a medical remedy, the herbs are often used in tea, soup and other foods in peoples’ daily lives. Therefore, people in the southern region have a higher poisoning risk from adulteration of their frequent use of custom TCMs. With the strongest support for the development of TCMs from the nation [9], a more modernized and effective technique of TCM identification is required to ensure safety in its use.

DNA barcoding is a novel technique using short and standard DNA regions to achieve accurate and rapid species identification [10]. This method can overcome the shortcoming of required intact diagnostic characters for morphological identification. Since herbal products are always traded in the form of dried, fragmented or powdered plant parts, including leaves, flowers, seeds, and roots, it is difficult to distinguish them by morphology. In addition, DNA barcoding is not affected by plant age or geographical variations that can influence some traditional identification methods, such as chemotaxonomy, chromatography, and microscopy. Therefore, DNA barcode is an ideal method for species identification, especially for herbal products. Some gene fragments from plasmids (mitochondrion & chloroplast) and nuclear genomes have been proposed to be barcodes since the concept was developed [10–12]. Unlike the standard mitochondrial cytochrome c oxidase I (COI) for animal barcoding, DNA barcoding in plants has persisted for a long time, and several barcodes have been investigated. Compared with the other six most often used DNA barcodes *psbA-trnH*, *matK*, *rbcL*, *rpoC1*, *ycf5* and ITS, Chen *et al*. [12] found that the second internal transcribed spacer (ITS2) of nuclear ribosomal DNA represents the most suitable region for DNA barcode applications. Along with ITS, ITS2 was recommended as one of the core plant barcodes by the China Plant BOL Group in 2011 [13]. Furthermore, Yao *et al*. [13] evaluated the ITS2 barcode from 50,790 plants and 12,221 animals and considered it a universal DNA barcode. As a standard DNA barcode for medicinal plant identification, the ITS2 barcode has been included for the first time in the Pharmacopoeia of the People’s Republic of China and its online Supplementary Note 2 for accurate TCM molecular identification [12]. An ITS2-based Chinese Materia Medica identification system was also developed [14].

Several DNA barcode reference libraries have been constructed, including fish fauna [15], invasive plants [16] and beetles [17], for a stronger, validated and more reliable identification purpose. Here, we sequenced the ITS2 barcode for constructing a library of southern herbs in China. This study can not only make up for the lack of a local barcode library of Southern Chinese Medicine but also improve the barcode database of TCM in China. Thus, it will help protect the health of people, especially people from the southern region. The main objectives of this study were (1) to construct a reliable reference DNA barcode library for southern herbs and (2) to evaluate the efficiency of species authentication of ITS2.

## Materials and methods

### Sample collection

**Fig 1:**
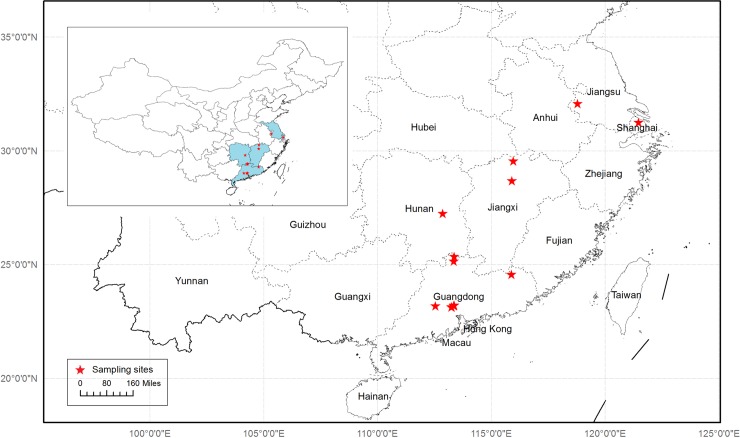
Sampling sites of medicinal plants in South China. Provinces of sampling sites are shadowed in the small map of China.

We collected fresh plant leaves from 1374 medicinal plant specimens in South China, mainly in Guangdong Province (Fig 1). The leaves were dried in silica gel as they were removed from the plants. Fieldwork was finished under the instructions and identification of a taxonomist, Hua-gu Ye, from the South China Botanical Garden, Chinese Academy of Sciences. Another 138 herbal product samples were purchased or obtained cost-free from the retailers KangMei Pharmaceutical (Puning, Guangdong Province) or Nanling Pharmaceutical (Meizhou, Guangdong Province) and identified by the chief traditional Chinese pharmacist Zhi-hai Huang at the Guangdong Provincial Hospital of Traditional Chinese Medicine. All voucher samples were deposited at the Guangdong Provincial Hospital of Traditional Chinese Medicine. A total of 1512 specimens were obtained from November 2015 to October 2016. More details about the collected samples are shown in S1 Table.

### DNA extraction, polymerase chain reaction and sequencing

Specimens were ground by a homogenization apparatus (Bertin Technologies, France) or liquid nitrogen. Genomic DNA was extracted from plant powders using a Plant Genomic DNA kit (Tiangen Biotech Beijing Co., China) according to the manufacturer’s instructions. The ITS2 barcodes were amplified on a ProFlex PCR system (Applied Biosystems, USA) in 25 μL reactions containing approximately 100 ng of template DNA, 12.5 μL of 2× PCR mix (0.05 units/μL Taq DNA polymerase, 4 mM MgCl2, and 0.4 mM dNTPs), 1.0 μL of each primer (2.5 μmol/L), and finally ddH_2_O to the final volume. Amplification was performed under the following conditions: 5 min at 94°C; 40 cycles of 30 s at 94°C, 30 s at 56°C, and 45 s at 72°C; 10 min at 72°C; held at 4°C. PCR amplicons were visualized on a 1.5% agarose gel and then cleaned and sequenced in both directions on an ABI3700xl capillary sequencer at the Majorbio Guangzhou branch office, China. The universal primer pair S2F (5’-ATGCGATACTTGGTGTGAAT-3’) and S3R (5’-GACGCTTCTCCAGACTACAAT-3’) was employed for PCR and Sanger sequencing reactions.

### Data analysis

Bidirectional sequences were assembled using CodonCode Aligner version 5.1.3 (CodonCode Corporation, USA) and annotated to the complete ITS2 by cutting off the conserved 5.8S and 28S motifs based on HMM [18] and the ITS2 database [19]. Sequence statistics (sequence length and nucleotide composition) were calculated in BOLD [20]. ITS2 barcode sequences were aligned; then, genetic distances within species, genera and families were calculated with the Kimura 2-Parameter (K2P) model [21] using the ‘Distance Summary’ sequence analysis tool of BOLD. The ‘Pairwise deletion’ option was chosen to treat gaps and missing data. Genetic distance-based methods (DNA barcoding gap and neighbor-joining tree) and similarity-based methods (BLAST) were used to evaluate the discrimination power of the ITS2 barcode. Barcoding gap analysis was carried out with the ‘Barcoding Gap Analysis’ tool in the BOLD system. A neighbor-joining tree was constructed with 1000 bootstrap replicates for all ITS2 barcodes in MEGA version 6.0 [22]. Basic Local Alignment Search Tool (BLAST) was performed against GenBank (http://blast.ncbi.nlm.nih.gov/Blast.cgi). All annotated ITS2 barcode sequences were submitted to BOLD, while the full length of sequences was uploaded to GenBank.

## Results

### Efficiency of PCR and sequence characterization

A total of 1512 specimens representing 359 species, 278 genera and 115 families were collected for analysis, with 138 herbal products and 1374 plants included (S1 Table). The samples consisted of 86.71% dicotyledons, 8.20% monocotyledons, 1.19% gymnosperms and 3.90% ferns. After amplification and sequencing, 1276 sequences were obtained with an 84.39% success rate in the total number of samples. Success rates were the highest in gymnosperms (100.00%) and the lowest (18.64%) in ferns (Table 1). These successful sequences were classified into 309 species, 243 genera and 100 families. The remaining 236 unsuccessful sequences were classified as 79 species, belonging to 49 families and 74 genera. Among them, 91 specimens failed to amplify the ITS2 barcode, and another 145 sequences had low quality. The efficiency of PCR in plants and herbal products was 84.43% and 84.06%, respectively, and no significant difference was observed as revealed by Chi-square (p). The success and failure in amplification or sequencing of different specimens always showed consistency in one species (93.8% of all species with more than one specimen). This result implied that primer binding or gene structure possibly played a more important role in sequence acquisition in this study. Finally, 187 barcodes of 54 new species were added to the BOLD system, and 123 new barcodes of 37 species were added to the GenBank database. The new species and barcodes are listed in S2 Table.

**Table 1:** PCR success rates of ITS2 in this study.

Taxa	No. of families	No. of genera	No. of species	No. of samples	No. of sequences	PCR Success rates
Dicotyledons	87	227	301	1311	1170	89.24%
Monocotyledons	15	35	38	124	77	62.10%
Gymnosperms	3	4	6	18	18	100.00%
Ferns	10	12	14	59	11	18.64%
Total	115	278	359	1512	1276	84.39%

**Fig 2:**
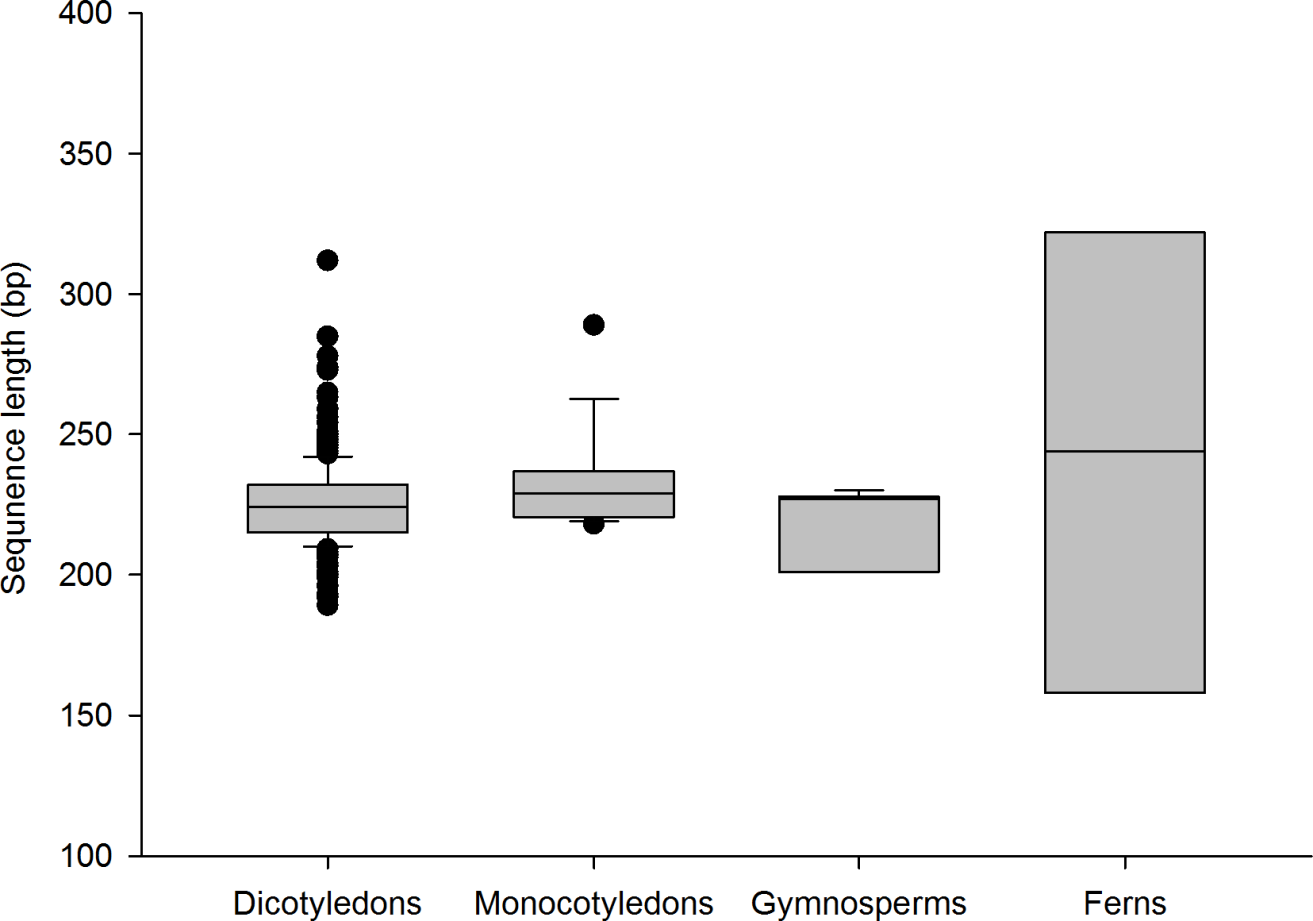
Box plots of the ITS2 barcodes length of southern herbs. The box plot illustrates the interquartile range (IQR) of the data. The IQR is defined as the difference between the 75th percentile and the 25th percentile. The line through the box represents the median length.

**Fig 3:**
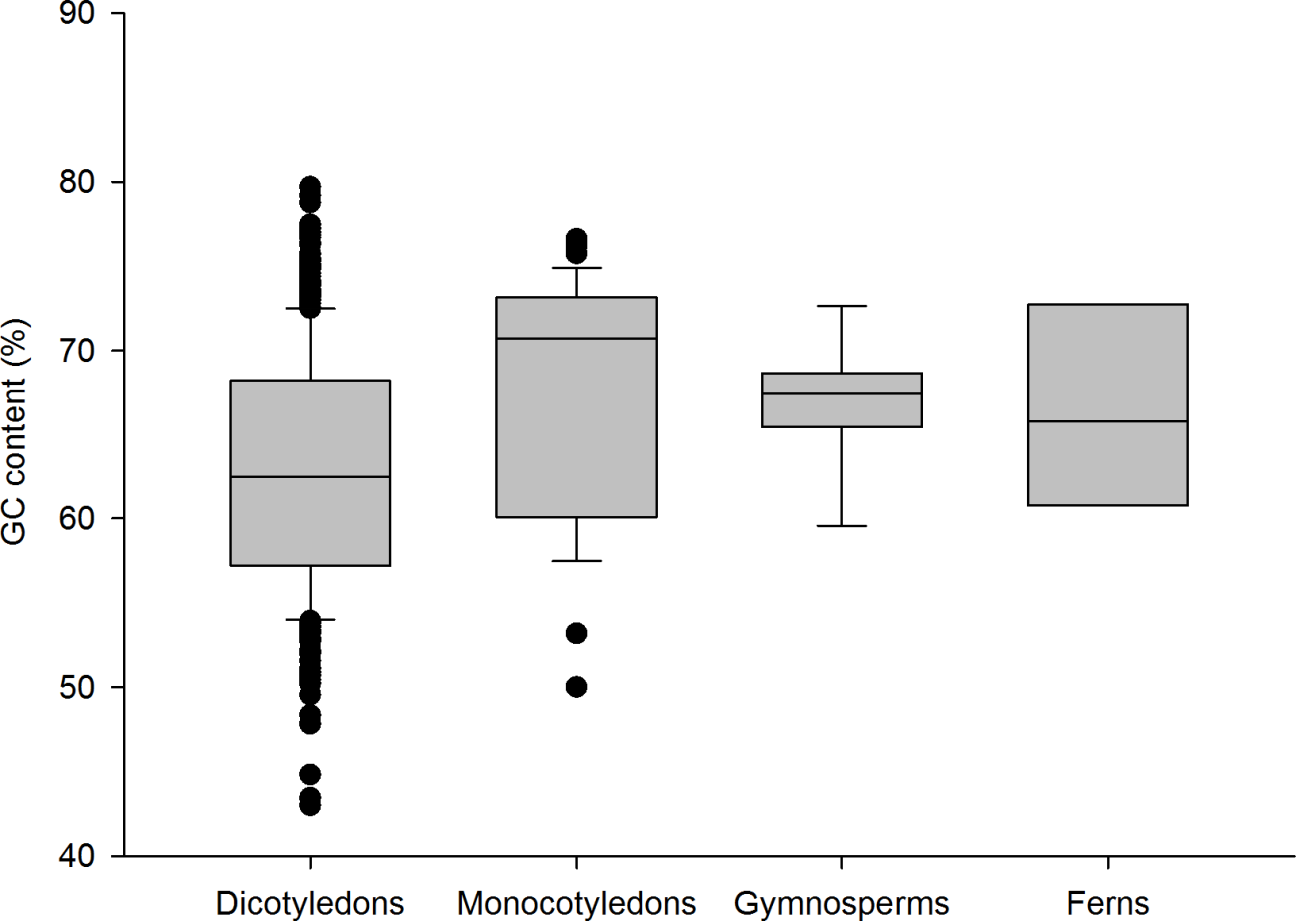
Box plots of the GC contents of ITS2 barcodes of southern herbs. The box plot illustrates the IQR of the data. The IQR is defined as the difference between the 75th percentile and the 25th percentile. The line through the box represents the median GC contents.

A full length sequence of nearly 500 bp was obtained with the Sanger sequencing method. Subsequent analyses were restricted to the annotated ITS2 sequence. With annotation, the length of all ITS2 barcodes varied between 158 bp and 348 bp, with an average length of 225 bp for all species. The average length of ITS2 in dicotyledons, monocotyledons, gymnosperms and ferns was 225 bp, 234 bp, 218 bp and 238 bp, respectively (Fig 2). The average nucleotide content was G = 30.53%, C = 32.70%, A = 16.71%, and T = 20.06%. The GC contents of all ITS2 sequences were 42.99%-79.69%, with an average of 63.23%. The average GC contents in the four taxa were 62.9%, 67.5%, 66.9% and 66.3% (Fig 3).

### Genetic divergence, DNA barcoding gap and Neighbor-Joining tree

**Fig 4:**
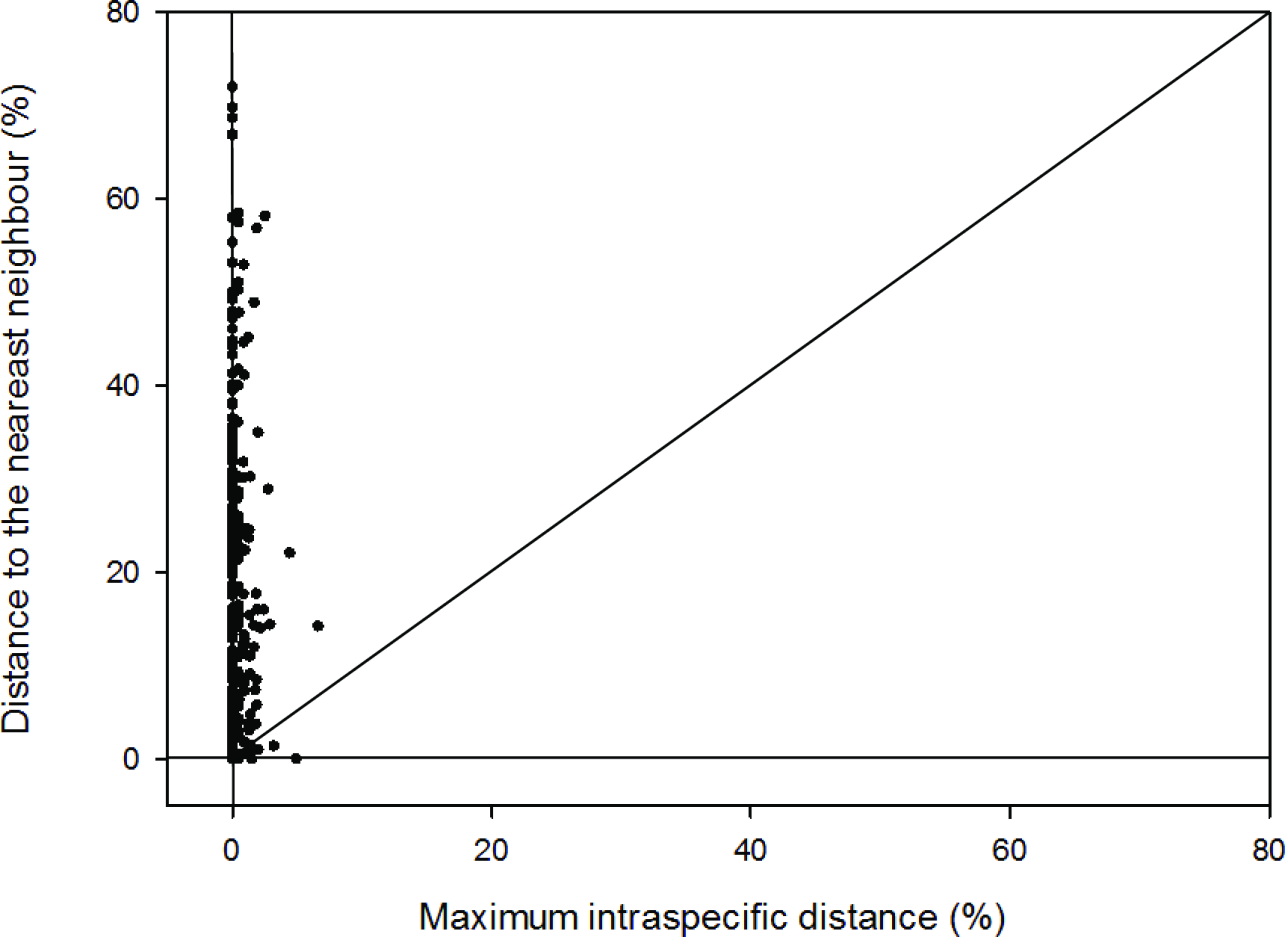
Scatter plots of maximum intraspecific K2P distance VS minimum distance to the nearest neighbor based on ITS2 barcodes.

Genetic distances increased as the taxon grew larger (Table 2). The within-species mean pairwise sequence divergence was 0.63%, with a minimum distance of 0.00% and a maximum distance of 44.53%. The mean distances at the genus and family levels were 11.77% and 36.98%, respectively. The Barcoding Gap Analysis (BGA) was demonstrated by plotting the maximum intra-specific genetic distance of each species against its minimum inter-specific distance (Fig 4). The former value was always lower than the latter except for five species listed in S3 Table. This result meant that ITS2 could not work for these species. Among them, two species pairs were original variants that always exceeded the application of DNA barcoding. The remaining three species pairs belonged to the same genus. They were *Alocasia macrorrhizos* & *Alocasia cucullata*, *Isodon serra* & *Isodon coetsa*, and *Melastoma malabathricum* & *Melastoma sanguineum*. Ten singleton species were excluded in the BGA analysis and NJ tree construction. The NJ tree formed 239 sequence clusters (S1 Fig). Among them, 202 clusters represented a single species, which meant that 67.56% of the species could be identified by the ITS2-based NJ tree. Another 97 species formed clusters and thus could not be distinguished in the NJ tree.

**Table 2:** Summary of K2P pairwise genetic distances (%) at different taxonomic levels.

Comparisons Within (Label)	Taxa	No. of Comparisons	Minimum Distance	Maximum Distance	Mean distance
Species	296	3276	0.00	44.53	0.63
Genus	49	2973	0.00	47.06	11.77
Family	38	23107	3.10	75.00	36.98

### BLAST analysis

BLAST identification was regarded as correct when query and best match sequences belonged to the same species, ambiguous when they were from both the same and different species, or incorrect when they were different species. New barcodes were excluded from the statistical analysis at the species level but included at the genus level with more than 95% BLAST identity.

Based on the similarity method, all 1153 specimens could be successfully identified at a success rate of 67.31% at the species level. All of the correctly identified species had a ≥97% similarity with the best match sequences except for the three species *Mucuna sempervirens* (96%), *Commelina communis* (95%), and *Cerbera manghas* (94%). Sequence coverage was always 98%–100%, with the exception of *Securinega virosa* (95%), *Liquidambar formosana* (95%), *Mytilaria laosensis* (95%), *Sauropus spatulifolius* (94%), *Rhodoleia championii* (93%), *Cerbera manghas* (86%) and *Bretschneidera sinensis* (73%). Regarding the genus level, the correct identification rate could reach as high as 96.57% (of 1223 specimens). In fact, the incorrect identification rate was relatively low, with 11.01% at the species level (species with incorrect identification are listed in S4 Table). The 21.68% ambiguous identification rate indicated that the ITS2 barcode could not tell them apart at the species level for some genera (Table 3), such as *Artemisia* (*Artemisia capillaries* and *Artemisia indica*), *Bidens* (*Bidens bipinnata*, *Bidens pilosa* and *Bidens tripartita*), *Euphorbia* (*Euphorbia hirta*, *Euphorbia humifusa* and *Euphorbia thymifolia*) and so on (S5 Table). In addition, conspecific individuals of 96% of species exhibited consistency in the BLAST result. The remaining 11 species showed cryptic diversity as different best match species were revealed for different query sequences of one species. They were *Dalbergia odorifera*, *Elsholtzia argyi*, *Gendarussa ventricosa*, *Glochidion wrightii*, *Ixora chinensis*, *Lantana camara*, *Litsea rotundifolia* var. *oblongifolia*, *Neolamarckia cadamba*, *Polygonum tinctorium*, *Potentilla chinensis* and *Syzygium samarangense*.

**Table 3:** Identification success rates of ITS2 through the BLAST method.

Comparison Levels	Correct identification (%)	Incorrect identification (%)	Ambiguous identification (%)
Species level	67.31%	11.01%	21.68%
Genus level	96.57%	0.65%	2.78%

A small portion of the identification was ambiguous (2.78%) or incorrect (0.65%) at the genus level (Table 3). Take *Schefflera octophylla* as an example, the best match species were *Macropanax dispermus*, *Brassaiopsis glomerulata*, and *Schefflera heptaphylla* from three genera of one family (S2 Fig). Incorrect sequences were discovered in the database during our BLAST process. For sequences of *Ageratum conyzoides*, the only best match sequence was from *Praxelis clematidea* (accession number KC012527) except for *Ageratum conyzoides* itself. To further test and verify, all ITS2 sequences of *Praxelis clematidea* were downloaded from GenBank and aligned together. We inferred from variation sites that this sequence was probably from *Ageratum conyzoides* instead of *Praxelis clematidea* (S3 Fig).

## Discussion

In this study, 1276 ITS2 barcode sequences of 1512 southern Chinese herbal samples were obtained. These ITS2 barcodes covered 309 species from 89 families and 224 genera. Genetic distance-based methods and similarity-based methods were employed to analyze the ITS2 identification power. We demonstrated the validity and efficiency of the ITS2 barcode for the identification of a wide range of southern plant species in China. Thus, a reliable reference ITS2 barcode library was constructed for Southern Chinese Medicine. It will be used for SCM quality monitoring and control to ensure its safety. However, we noticed that there is a discrepancy among the results from the three different methods, BGA, NJ tree and BLAST. Five species had a greater maximum intraspecific K2P distance than the minimum distance to the nearest neighbor in BGA. Thirty-five species were incorrectly identified, and sixty-eight species were ambiguously identified by BLAST analysis. Ninety-seven species could not be identified by the ITS2-based NJ tree. In theory, species falling below the diagonal in BGA could be misidentified with their nearest neighbors by K2P and BLAST, and these species should not be recognized by the NJ tree. Although five species and their nearest species were in one clade in the NJ tree, they were not misidentified by BLAST. In fact, only two of them were found to be ambiguously (*Rubus reflexus* var. *lanceolobus*) or incorrectly identified (*Melastoma malabathricum*). Even so, the nearest species were not the query’s best-matched sequences. For species incorrectly or ambiguously identified by BLAST, 41.94% and 40.00% of them failed to be identified by the NJ tree, respectively. That meant that no more than half of the species were identified with the same results by these two methods. The main reason was that the BGA and NJ tree evaluated the barcodes in the reference library alone, while BLAST compared the barcodes to those in the nucleotide database of NCBI. The different scope of the research along with the imperfection of the dataset and limitation of the methods all contributed to the identification results [23].

ITS2 is first described by Chiou *et al*. [24] and is often used as a phylogenetic marker in evolutionary analyses [25–27]. Based on extensive studies, ITS2 has been one of the most promising DNA barcodes for plants and has been applied to many types of medicinal plant for identification [6,28]. The ITS2 barcode has obvious advantages. First, ITS2 has high amplification efficiency. In this study, we successfully amplified and sequenced 84.39% of all 1512 specimens. According to Chen *et al*. [12], the success rate for ITS2 was 93.8%. Conserved regions of ITS2 enable the design of primers with good universality to obtain positive amplification. No other potential barcode exhibits such high universality as ITS2. Second, the length of the ITS2 barcode is short. The ITS2 barcode length of plants was mainly distributed in the 158–348 bp range in our study. Almost all ITS2 barcodes studied so far are less than 500 bp in length. The short gene length not only makes PCR and sequencing easier but also makes it especially important for gaining sequences of herbal products containing genomic DNA with a certain degree of degradation. Furthermore, the short gene length allows ITS2 to be applied in high-throughput sequencing that could simultaneously amplify several thousand 100–200 bp DNA molecules in one reaction. Thus, we can identify thousands of species at a time. Third, the ITS2 barcode has strong species resolving power. Here, ITS2 could successfully identify nearly 70% of specimens at the species level by NJ tree and BLAST analysis. According to Xu *et al*. [16], ITS2 has the greatest inter-specific variation compared to *trnH-psbA*, ITS, *rbcL*, and *matK*. In addition, the secondary structures of ITS2 would add additional information to help improve discrimination between species [29–31]. Therefore, the benefits of using the ITS2 barcode to identify species may be twofold. As a nuclear gene inherited from both parents, ITS2 provides more information than organellar DNA and is likely to have broader taxonomic applicability. Although ITS2 has many advantages, it does not work as well in some taxa of ferns. Low PCR efficiency has become an obvious shortcoming based on our study and other studies [12, 32]. Heterogeneity is also a problematic issue for ITS2 as a consequence of concerted evolution [25]. However, greater advantages make ITS2 an excellent barcode candidate.

The global trade of TCM has experienced explosive growth in recent years, and the market for herbal products seems to have a brighter future. During the period of industrial prosperity, authentic herbs would be sold to the public mixed with adulterants of less expensive and less effective, even dangerous, herbs. Accurate and fast methods for species identification of DNA barcoding precede other traditional methods. The application of DNA barcoding for species identification strongly depends on the quality of reference sequences. Unfortunately, sequence information available in public libraries is often missing, resulting from inadequate and insufficient records. The missing information might lead to ambiguous results that possess a relatively high proportion (21.68% of all) in our study. Incorrect results, along with some of the ambiguous ones, are possibly from either morphological misidentifications of voucher samples, contamination during lab procedures, or synonym names [33]. Robust DNA barcode reference libraries are consequently critical. Although DNA barcoding has many advantages, it cannot completely replace traditional methods. For processing drugs where genomic DNA is severely damaged, the DNA barcoding technique cannot work. Therefore, we should combine DNA barcoding with biochemical and morphological traits to facilitate a better and safer utilization of TCM resources.

## Conclusions

We sequenced ITS2 of Southern herbs in China and constructed the first local reference barcode library for Southern Chinese Medicine. This barcode library will facilitate SCM product quality control, thus contributing to protecting public health.

## Supporting information

S1 Fig NJ tree based on all the ITS2 barcode sequences of southern herbs in this study. (PDF)

S2 Fig NJ tree based on ITS2 sequences of *S. heptaphylla*, *M. dispermus* and *B. glomerulata*. Different sequences of one species are in the same color.
(PDF)

S3 Fig ITS2 alignment analysis of *A. conyzoides* and *P. clematidea*. The distinctive sequence of *Praxelis clematidea* is outlined.
(PDF)

S1 Table Sequence details with voucher sample information, GenBank accession numbers and BOLD processed IDs in this study. (XLSX)

S2 Table New barcodes added to BOLD and GenBank. (DOCX)

S3 Table Species with a greater maximum intraspecific K2P distance than the minimum distance to the nearest neighbor based on ITS2. (DOCX)

S4 Table Incorrect identification at the species level by BLAST analysis based on the ITS2 barcode. (DOCX)

S5 Table Ambiguous identification at the species level by BLAST analysis based on the ITS2 barcode. (DOCX)
