# Supplementary figures and images for: Constructing a DNA barcode reference library for southern herbs in China: A resource for authentication of southern Chinese medicine
Source: PLoS One. 2018 Jul 25;13(7):e0201240. doi: 10.1371/journal.pone.0201240 (PMC6059470; doi:10.1371/journal.pone.0201240)

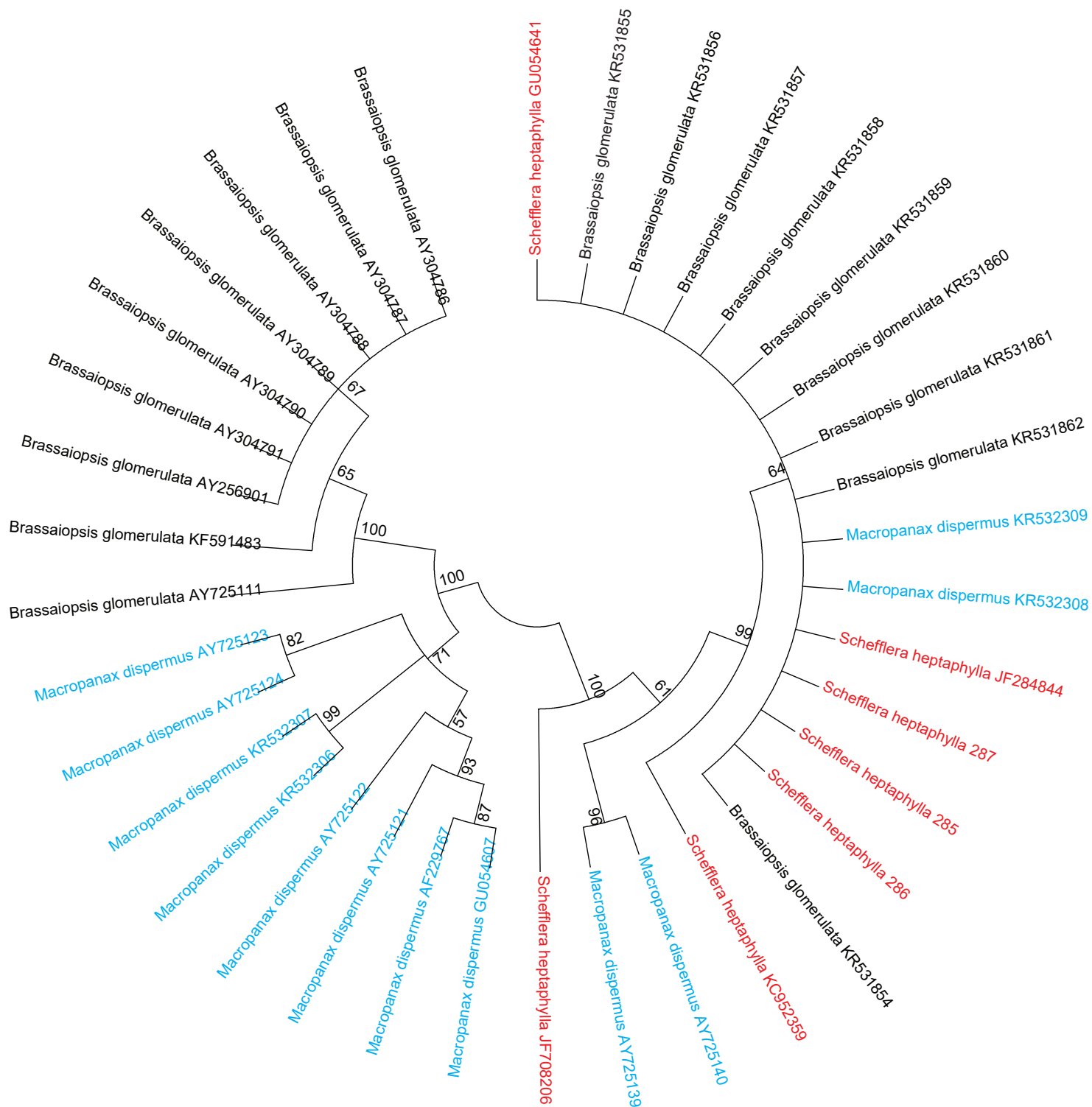

Supplement: S2 Fig — Different sequences of one species are in the same color. (PDF) [file pone.0201240.s002.pdf]
