# Supplementary material for: Constructing a DNA barcode reference library for southern herbs in China: A resource for authentication of southern Chinese medicine
Source: PLoS One. 2018 Jul 25;13(7):e0201240. doi: 10.1371/journal.pone.0201240 (PMC6059470; doi:10.1371/journal.pone.0201240)
Supplement: S3 Fig — The distinctive sequence of Praxelis clematidea is outlined. (PDF) [file pone.0201240.s003.pdf]

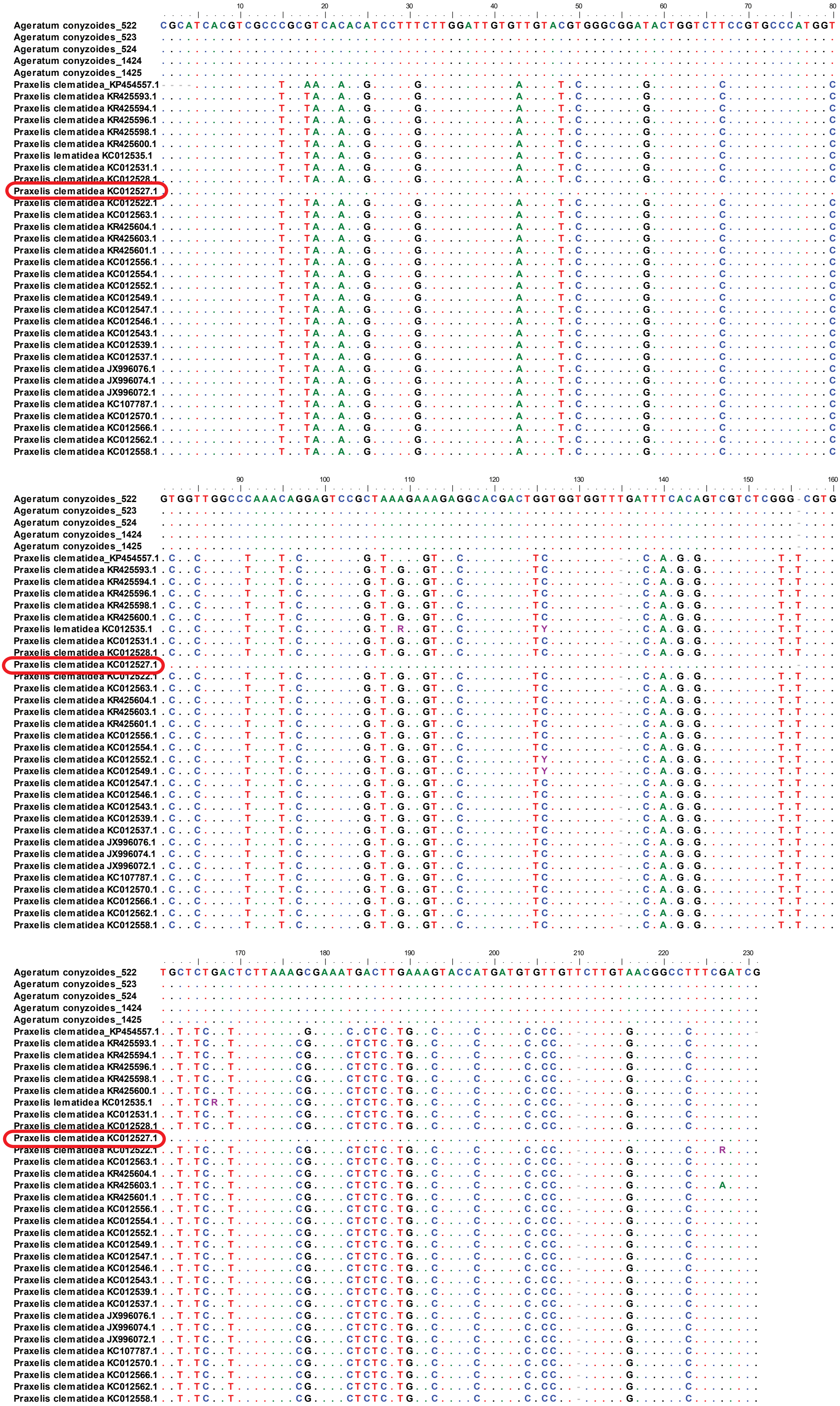
Ageratum conyzoides_522 CGCATCACGTCGCCCGCGTCACACATCCTTTCTTGGATTGTGTTGTACGTGGGCGGATACTGGTCTTCCGTGCCCATGGT
Ageratum conyzoides_522 GTGGTTGGCCCAAACAGGAGTCCGCTAAAGAAAGAGGCACGACTGGTGGTGGTTTGATTTCACAGTCGTCTCGGG-CGTG
Ageratum conyzoides_522 TGCTCTGACTCTTAAAGCGAAATGACTTGAAAGTACCATGATGTGTTGTTCTTGTAACGGCCTTTCGATCG
Ageratum conyzoides_523
Ageratum conyzoides_524
Ageratum conyzoides_1424
Ageratum conyzoides_1425
Praxelis clematidea_KP454557.1
Praxelis clematidea KR425593.1
Praxelis clematidea KR425594.1
Praxelis clematidea KR425596.1
Praxelis clematidea KR425598.1
Praxelis clematidea KR425600.1
Praxelis lematidea KC012535.1
Praxelis clematidea KC012531.1
Praxelis clematidea KC012528.1
Praxelis clematidea KC012527.1
Praxelis clematidea KC012522.1
Praxelis clematidea KC012563.1
Praxelis clematidea KR425604.1
Praxelis clematidea KR425603.1
Praxelis clematidea KR425601.1
Praxelis clematidea KC012556.1
Praxelis clematidea KC012554.1
Praxelis clematidea KC012552.1
Praxelis clematidea KC012549.1
Praxelis clematidea KC012547.1
Praxelis clematidea KC012546.1
Praxelis clematidea KC012543.1
Praxelis clematidea KC012539.1
Praxelis clematidea KC012537.1
Praxelis clematidea JX996076.1
Praxelis clematidea JX996074.1
Praxelis clematidea JX996072.1
Praxelis clematidea KC107787.1
Praxelis clematidea KC012570.1
Praxelis clematidea KC012566.1
Praxelis clematidea KC012562.1
Praxelis clematidea KC012558.1
