# Supplementary material for: Constructing a DNA barcode reference library for southern herbs in China: A resource for authentication of southern Chinese medicine
Source: PLoS One. 2018 Jul 25;13(7):e0201240. doi: 10.1371/journal.pone.0201240 (PMC6059470; doi:10.1371/journal.pone.0201240)
Supplement: S2 Table — (DOCX) [file pone.0201240.s005.docx]

**Table S2 New barcodes added to BOLD and GenBank.**

| Species | GenBank Accession NO. | BOLD ID |
| --- | --- | --- |
| *Acalypha australis* |  | LNMP238-17, LNMP239-17, LNMP240-17 |
| *Achyranthes longifolia* | MG730611, MG730612, MG730613, MG730614, MG730615 | LNMP131-17, LNMP132-17, LNMP133-17, LNMP134-17, LNMP135-17 |
| *Adenosma glutinosum* |  | LNMP1113-17 |
| *Allemanda neriifolia* | MG730449, MG73050 |  |
| *Ardisia alyxiaefolia* | MG730844, MG730845, MG730846 | LNMP511-17, LNMP512-17, LNMP513-17 |
| *Ardisia gigantifolia* | MG731200, MG731201, MG731202 | LNMP520-17, LNMP521-17, LNMP522-17 |
| *Asystasiella neesiana* |  | LNMP965-17, LNMP966-17, LNMP967-17 |
| *Bauhinia championii* |  | LNMP624-17, LNMP625-17, LNMP626-17 |
| *Blumea formosana* |  | LNMP070-17, LNMP071-17, LNMP072-17 |
| *Bowringia callicarpa* |  | LNMP677-17, LNMP678-17, LNMP679-17 |
| *Buxus sinica* |  | LNMP956-17, LNMP957-17 |
| *Campanumoea javanica* |  | LNMP005-17, LNMP006-17 |
| *Cayratia corniculata* | MG730509, MG730510, MG730511 | LNMP596-17, LNMP597-17, LNMP598-17 |
| *Chrysanthemum indicum* |  | LNMP076-17, LNMP077-17, LNMP078-17 |
| *Clematis filamentosa* | MG730336, MG730337, MG730338, MG730339, MG730340 | LNMP591-17, LNMP592-17, LNMP593-17, LNMP594-17, LNMP595-17 |
| *Clerodendrum hainanense* | MG730383, MG730384, MG730385 | LNMP1146-17, LNMP1147-17, LNMP1148-17 |
| *Craibiodendron scleranthum* |  | LNMP229-17, LNMP230-17, LNMP231-17 |
| *Cryptomeria fortunei* | MG730608, MG730609, MG730610 |  |
| *Daphniphyllum calycinum* |  | LNMP235-17, LNMP236-17, LNMP237-17 |
| *Dieffenbachia seguine* | MG730427, MG730428, MG730429 |  |
| *Dinetus racemosus* | MG730314, MG730315, MG730316 |  |
| *Ehretia microphylla* | MG730472, MG730473, MG730474, MG730475 |  |
| *Elephantopus tomentosus* | MG730089, MG730090, MG730091, MG730092, MG730093, MG730094,  MG730095, MG730096 | LNMP089-17, LNMP090-17, LNMP091-17, LNMP092-17, LNMP093-17, LNMP094-17,  LNMP095-17, LNMP096-17 |
| *Elsholtzia argyi* |  | LNMP1028-17, LNMP1029-17, LNMP1030-17, LNMP1031-17 |
| *Embelia laeta* |  | LNMP526-17, LNMP527-17, LNMP528-17, LNMP529-17, LNMP530-17 |
| *Eragrostis atrovirens* | MG730868, MG730869, MG730870 | LNMP1250-17, LNMP1251-17, LNMP1252-17 |
| *Erycibe obtusifolia* |  | LNMP1005-17, LNMP1006-17, LNMP1007-17 |
| *Eucalyptus robusta* | MG730216, MG730217, MG730218 |  |
| *Tetradium glabrifolium* |  | LNMP915-17, LNMP916-17, LNMP917-17 |
| *Evodia lepta* |  | LNMP101-17, LNMP102-17, LNMP103-17, LNMP104-17, LNMP105-17 |
| *Tetradium austrosinense* |  | LNMP918-17, LNMP919-17, LNMP920-17, LNMP921-17, LNMP922-17 |
| *Falcataria moluccana* | MG751359, MG751360, MG751361 |  |
| *Justicia ventricosa* |  | LNMP973-17, LNMP974-17, LNMP975-17,  LNMP976-17, LNMP977-17,LNMP978-17 |
| *Glochidion zeylanicum* var. *tomentosum* | MG730418 |  |
| *Gynura divaricata* | MG730122, MG730123, MG730124 | LNMP106-17, LNMP107-17, LNMP108-17 |
| *Hibiscus tiliaceus* | MG730454, MG730455, MG730456 |  |
| *Hydrocotyle nepalensis* |  | LNMP1176-17, LNMP1177-17, LNMP1178-17 |
| *Hygrophila salicifolia* |  | LNMP979-17, LNMP980-17, LNMP981-17, LNMP982-17, LNMP983-17, LNMP984-17 |
| *Isodon amethystoides* |  | LNMP1073-17, LNMP1074-17, LNMP1075-17 |
| *Isoglossa collina* |  | LNMP985-17, LNMP986-17, LNMP987-17 |
| *Itea omeiensis* | MG730536, MG730537, MG730538 |  |
| *Lindernia ruellioides* | MG730396, MG730397, MG730398 |  |
| *Livistona chinensis* | MG730782, MG730783 |  |
| *Lobelia melliana* |  | LNMP196-17, LNMP197-17, LNMP198-17 |
| *Ludwigia octovalvis* | MG730677, MG730678, MG730679 |  |
| *Lycianthes biflora* | MG730853, MG730854, MG730855 |  |
| *Lycoris aurea* |  | LNMP1269-17, LNMP1270-17, LNMP1271-17 |
| *Lygodium scandens* |  | LNMP1332-17, LNMP1333-17, LNMP1334-17 |
| *Maesa perlarius* | MG730477, MG730478, MG730479, MG730480, MG730481, MG730482 |  |
| *Mahonia bealei* |  | LNMP551-17, LNMP552-17, LNMP553-17, LNMP554-17 |
| *Melastoma sanguineum* | MG730689, MG730690, MG730691, MG730692, MG730692 | LNMP400-17, LNMP401-17, LNMP402-17, LNMP403-17, LNMP404-17 |
| *Mimosa pudica* | MG730395 |  |
| *Mosla scabra* |  | LNMP1060-17, LNMP1061-17, LNMP1062-17 |
| *Mucuna sempervirens* |  | LNMP702-17, LNMP703-17, LNMP704-17 |
| *Mussaenda erosa* |  | LNMP846-17, LNMP847-17, LNMP848-17 |
| *Odontosoria chusana* | MG730965, MG730966 | LNMP1330-17, LNMP1331-17 |
| *Ophiorrhiza cantonensis* | MG730362, MG730363, MG730364 |  |
| *Oreocnide frutescens* |  | LNMP1218-17, LNMP1219-17 |
| *Parthenocissus dalzielii* | MG731099, MG731100 | LNMP600-17, LNMP601-17 |
| *Dacrycarpus imbricatus var. patulus* |  | LNMP1311-17, LNMP1312-17, LNMP1313-17 |
| *Polycarpaea corymbosa* | MG730076, MG730077, MG730078, MG730079, MG730080, MG730081,  MG730082, MG730083, MG730084, MG730085 | LNMP151-17, LNMP152-17, LNMP153-17, LNMP154-17, LNMP155-17, LNMP156-17,  LNMP157-17, LNMP158-17, LNMP159-17, LNMP160-17 |
| *Pueraria phaseoloides* | MG730810, MG730811, MG730812 |  |
| *Pueraria lobata* var. *montana* |  | LNMP705-17, LNMP706-17, LNMP707-17 |
| *Rubus chingii* var. *suavissimus* | MG730920, MG730921, MG730922 |  |
| *Rubus reflexus* var. *lanceolobus* |  | LNMP772-17, LNMP773-17, LNMP774-17 |
| *Sabia discolor* | MG730058, MG730059, MG730060 | LNMP958-17, LNMP959-17, LNMP960-17 |
| *Sabia japonica* | MG730801 |  |
| *Sapium sebiferum* |  | LNMP328-17, LNMP329-17, LNMP330-17, LNMP331-17 |
| *Saurauia tristyla* |  | LNMP461-17, LNMP462-17, LNMP463-17, LNMP464-17 |
| *Serratula chinensis* |  | LNMP112-17, LNMP113-17, LNMP114-17 |
| *Setaria plicata* | MG731150, MG731151, MG731152 |  |
| *Stauntonia chinensis* |  | LNMP555-17 |
| *Struthiopteris eburnea* | MG757296, MG757297, MG757298 | LNMP434-17, LNMP435-17, LNMP436-17 |
| *Tetrastigma hemsleyanum* |  | LNMP602-17, LNMP603-17, LNMP604-17 |
| *Triumfetta pilosa* | MG730183, MG730184, MG730185 | LNMP377-17, LNMP378-17, LNMP379-17 |
