# Supplementary material for: Constructing a DNA barcode reference library for southern herbs in China: A resource for authentication of southern Chinese medicine
Source: PLoS One. 2018 Jul 25;13(7):e0201240. doi: 10.1371/journal.pone.0201240 (PMC6059470; doi:10.1371/journal.pone.0201240)
Supplement: S3 Table — (DOCX) [file pone.0201240.s006.docx]

**Table S3 Species with a greater maximum intraspecific K2P distance than the minimum distance to the nearest neighbor based on ITS2.**

| Species | Max Intra-Sp | Distance to NN | Nearest Species |
| --- | --- | --- | --- |
| *Alocasia macrorrhizos* | 1.4 | 0.35 | *Alocasia cucullata* |
| *Isodon lophanthoides* | 3.21 | 1.36 | *Isodon lophanthoides* var. *graciliflorus* |
| *Isodon serra* | 0.95 | 0.47 | *Isodon coetsa* |
| *Melastoma malabathricum* | 0.45 | 0 | *Melastoma sanguineum* |
| *Rubus reflexus* var. *lanceolobus* | 1.91 | 0.95 | *Rubus reflexus* |
