# Supplementary material for: Constructing a DNA barcode reference library for southern herbs in China: A resource for authentication of southern Chinese medicine
Source: PLoS One. 2018 Jul 25;13(7):e0201240. doi: 10.1371/journal.pone.0201240 (PMC6059470; doi:10.1371/journal.pone.0201240)
Supplement: S4 Table — (DOCX) [file pone.0201240.s007.docx]

**Table S4 Incorrect identification at the species level by BLAST analysis based on the ITS2 barcode.**

| No. | Species | Sample ID |
| --- | --- | --- |
| 1 | *Alpinia chinensis* | 1059-1061 |
| 2 | *Ardisia quinquegona* | 925-927 |
| 3 | *Astilbe grandis* | 66,453-455 |
| 4 | *Castanopsis hystrix* | 837-839 |
| 5 | *Coix lacryma-jobi* | 237-239,1421 |
| 6 | *Dianella ensifolia* | 705-707 |
| 7 | *Dalbergia odorifera* | 528 |
| 8 | *Embelia laeta* | 711-713,1545-1547 |
| 9 | *Ficus esquiroliana* | 970-972 |
| 10 | *Ficus variegata* | 1079-1081 |
| 11 | *Ilex latifolia* | 579-581,1259,1447 |
| 12 | *Kadsura longipedunculata* | 54-56,411-413,1190-1-1190-10 |
| 13 | *Litsea rotundifolia* var. *oblongifolia* | 552-554, 1466-1467 |
| 14 | *Lycoris aurea* | 294-296 |
| 15 | *Lycoris radiata* | 495-497 |
| 16 | *Melaleuca leucadendra* | 252-254, 1474-1475 |
| 17 | *Melastoma malabathricum* | 729-731, 1486-1487 |
| 18 | *Melastoma sanguineum* | 687-689,1490-1491 |
| 19 | *Mosla scabra* | 858-860 |
| 20 | *Musa ×paradisiaca* | 282-284 |
| 21 | *Myrica rubra* | 946-948 |
| 22 | *Paraphlomis javanica* | 447-449 |
| 23 | *Patrinia villosa* | 396-398 |
| 24 | *Persicaria tinctoria* | 1215 |
| 25 | *Piper austrosinense* | 585-587 |
| 26 | *Piper hancei* | 483-485 |
| 27 | *Potentilla chinensis* | 1161 |
| 28 | *Rubus reflexus* var. *lanceolobus* | 744-746 |
| 29 | *Stachyurus chinensis* | 369-371 |
| 30 | *Stauntonia chinensis* | 67 |
| 31 | *Stephania longa* | 732-734,1470-1471 |
| 32 | *Syzygium cumini* | 645-647 |
| 33 | *Syzygium samarangense* | 1104-1105 |
| 34 | *Tetradium austrosinense* | 68, 372-374, 1367 |
| 35 | *Zinnia elegans* | 474-476 |
