# Supplementary material for: Constructing a DNA barcode reference library for southern herbs in China: A resource for authentication of southern Chinese medicine
Source: PLoS One. 2018 Jul 25;13(7):e0201240. doi: 10.1371/journal.pone.0201240 (PMC6059470; doi:10.1371/journal.pone.0201240)
Supplement: S5 Table — (DOCX) [file pone.0201240.s008.docx]

**Table S5 Ambiguous identification at the species level by BLAST analysis based on the ITS2 barcode.**

| No. | Species | Sample ID |
| --- | --- | --- |
| 1 | *Ajuga ciliata* | 357-359 |
| 2 | *Alchornea trewioides* | 803-805,1462-1463 |
| 3 | *Amaranthus viridis* | 639-641 |
| 4 | *Anisomeles indica* | 101-103, 1330-1331 |
| 5 | *Aquilaria sinensis* | 1342,1500-1501 |
| 6 | *Artemisia capillaris* | 498-500 |
| 7 | *Artemisia indica* | 648-650,1396-1397,1588 |
| 8 | *Averrhoa carambola* | 660-662 |
| 9 | *Bauhinia variegata* | 690-692 |
| 10 | *Bidens pilosa* | 107-109,146-148,1147,1288，1596,1603-1605 |
| 11 | *Bidens tripartita* | 1145-1146 |
| 12 | *Blumea formosana* | 348-350 |
| 13 | *Boehmeria tricuspis* | 378-380 |
| 14 | *Buxus sinica* | 79-81 |
| 15 | *Cajanus cajan* | 952-954,1563-1566 |
| 16 | *Campanumoea javanica* | 486-488 |
| 17 | *Canarium album* | 1003-1005 |
| 18 | *Chrysanthemum indicum* | 465-467 |
| 19 | *Cinnamomum burmannii* | 546-548,1554-1556 |
| 20 | *Clinopodium chinense* | 402-404 |
| 21 | *Cynanchum auriculatum* | 501-503 |
| 22 | *Daphniphyllum calycinum* | 828-830 |
| 23 | *Dendropanax proteus* | 432-434 |
| 24 | *Dendrotrophe varians* | 870-872 |
| 25 | *Elsholtzia argyi* | 61 |
| 26 | *Eupatorium chinense* | 351-353,1179 |
| 27 | *Euphorbia hirta* | 188-190,1597 |
| 28 | *Euphorbia humifusa* | 606-608,1364 |
| 29 | *Euphorbia thymifolia* | 624-626 |
| 30 | *Eurya chinensis* | 531-533 |
| 31 | *Glochidion lanceolarium* | 1042-1043 |
| 32 | *Glochidion wrightii* | 76, 959 |
| 33 | *Goodyera schlechtendaliana* | 381-383 |
| 34 | *Gynostemma pentaphyllum* | 60,1594-1595 |
| 35 | *Hygrophila ringens* | 831-833, 1426-1428 |
| 36 | *Hymenocallis littoralis* | 159-160, 1443-1444 |
| 37 | *Hypericum monogynum* | 720-722 |
| 38 | *Ilex pubescens* | 819-821 |
| 39 | *Isatis tinctoria* | 1130, 1132-1138 |
| 40 | *Isodon amethystoides* | 861-863 |
| 41 | *Justicia ventricosa* | 603, 605, 1317 |
| 42 | *Lantana camara* | 113-115, 1600 |
| 43 | *Ligustrum sinense* | 573-575 |
| 44 | *Lonicera japonica* | 1612,1627-1628 |
| 45 | *Macaranga tanarius* | 1106-1108 |
| 46 | *Mahonia bealei* | 59,480-482 |
| 47 | *Mallotus paniculatus* | 855-857 |
| 48 | *Melastoma candidum* | 507-509 |
| 49 | *Mussaenda erosa* | 907-909 |
| 50 | *Nasturtium officinale* | 324-326 |
| 51 | *Neolamarckia cadamba* | 609 |
| 52 | *Oldenlandia herbacea* | 1148-1149, 1149-2, 1363 |
| 53 | *Oreocnide frutescens* | 922,924 |
| 54 | *Nageia nagi* | 167-169, 1464-1465 |
| 55 | *Potentilla discolor* | 1142-1-1142-11,1153-1-1153-10 |
| 56 | *Potentilla chinensis* | 1164 |
| 57 | *Psychotria rubra* | 543-545,1313,1567,1574-1575 |
| 58 | *Isodon lophanthoides* var. *graciliflorus* | 89-91 |
| 59 | *Ixora chinensis* | 876, 1482-1483 |
| 60 | *Rhododendron mariae* | 1053-1055 |
| 61 | *Rubus reflexus* | 492-494 |
| 62 | *Schefflera heptaphylla* | 285-287 |
| 63 | *Solanum nigrum* | 57-58,360-362,1373 |
| 64 | *Spatholobus suberectus* | 134-136,1608-1609 |
| 65 | *Acmella paniculata* | 840-842 |
| 66 | *Taxus wallichiana* var. *chinensis* | 1384 |
| 67 | *Taxus wallichiana var. mairei* | 663-665 |
| 68 | *Tetrastigma hemsleyanum* | 994-996 |
